# Migratory Deposition of Calcium Pyrophosphate in an Older Patient With Several Femoral Neck Implant Infection Episodes: A Case Report

**DOI:** 10.7759/cureus.50815

**Published:** 2023-12-20

**Authors:** Ryuichi Ohta, Tomohiro Shigetaka, Chiaki Sano

**Affiliations:** 1 Community Care, Unnan City Hospital, Unnan, JPN; 2 Orthopedic Surgery, Unnan City Hospital, Unnan, JPN; 3 Community Medicine Management, Shimane University Faculty of Medicine, Izumo, JPN

**Keywords:** orthopedic surgical procedures, diagnostic challenges, geriatrics, pseudogout, postoperative complications, methicillin-resistant staphylococcus aureus

## Abstract

This case report describes an 86-year-old female patient who presented with complex post-surgical complications following orthopedic surgery for a femoral neck fracture. Initially diagnosed with septic shock due to methicillin-resistant *Staphylococcus aureus* (MRSA) bacteremia at the surgical site, the patient's treatment course was complicated, involving multiple hospital transfers and varying treatments, including antibiotics and surgical drainage. Despite the absence of infection indicators post treatment, the patient later developed severe thigh pain and was found to have migratory pseudogout, an unusual diagnosis in the context of MRSA and post-surgical complications. This report emphasizes the diagnostic challenges in distinguishing between surgical site infections and other inflammatory conditions like migratory pseudogout, particularly in older patients with comorbidities. It underscores the importance of comprehensive evaluations and the need for general physicians to maintain a broad differential diagnosis when managing post-surgical infections. The case highlights the persistence and recurrence risk of MRSA infections, even post-appropriate antibiotic therapy, and the necessity of considering migratory pseudogout in patients with recurrent infections and systemic soft tissue involvement. The insights from this case contribute to the understanding of complex post-surgical complications and advocate for meticulous assessment and tailored treatment strategies in similar clinical scenarios.

## Introduction

Methicillin-resistant *Staphylococcus aureus* (MRSA) poses significant challenges in post-surgical infection management, particularly in diagnosis and treatment [1]. These challenges are exacerbated in older patients, who often have multiple comorbidities that complicate clinical presentation and treatment response [2]. Our case report details an older patient who, after orthopedic surgery, developed multiple surgical site infections attributed to MRSA but was ultimately diagnosed with migratory pseudogout. As the prevalence is unknown, this diagnosis is rare, especially in the context of post-surgical infections and MRSA, lending significance to this case [3].

The co-occurrence of MRSA infection and migratory pseudogout in this patient underscores the complexities in diagnosing and treating post-surgical complications in the elderly [4]. Migratory pseudogout, particularly outside joint manifestations, has been sparsely reported [5,6]. In our case, an older patient experienced migratory pseudogout amidst repeated MRSA infections at orthopedic surgical sites. This case emphasizes the need for a broad differential diagnosis in persistent or recurrent surgical site infections. It also highlights the difficulty in distinguishing infectious causes from inflammatory conditions like gout, pseudogout, and rheumatic diseases, which can present similarly to infections. The insights gained from this case are invaluable, especially for general physicians who often initially manage patients with such complications.

## Case presentation

An 86-year-old female patient was admitted to a rural community hospital for rehabilitation. One month prior, she had undergone surgery for a right femoral neck fracture resulting from a fall. Post surgery, she developed a fever and hypotension and lost consciousness, leading to a diagnosis of septic shock due to MRSA bacteremia at the surgical site. Following intensive treatment at a tertiary hospital, she was transferred to our facility without signs of infection. Her medical history included hypertension, diabetes mellitus, and dyslipidemia, treated with valsartan (80 mg daily), metformin (1000 mg daily), atorvastatin (5 mg daily), and minocycline (200 mg daily).

The vital signs at the visit were as follows: blood pressure, 124/78 mmHg; pulse rate, 68 beats/min; body temperature, 36.7°C; respiratory rate, 18 breaths/min; and oxygen saturation, 98% on room air. The patient was alert to time, place, and person. Physical examination, including the right femoral surgical site, revealed no abnormalities. She was moved to the rehabilitation unit on the seventh day. On day 28, minocycline was discontinued following a six-week course for the right femoral device infection.

On the 42nd day, she reported severe pain in her right thigh, rated 7/10, during rehabilitation. Examination revealed significant tenderness in the lateral right thigh. Laboratory tests indicated elevated inflammatory markers (Table 1).

**Table 1:** Laboratory data of the patient Na: sodium; K: potassium; Cl: chloride; CA: calcium; eGFR: estimated glomerular filtration rate; CK: creatine kinase; CRP: C-reactive protein; SARS-CoV-2: severe acute respiratory syndrome coronavirus 2

Marker	Level	Reference
White blood cells	6.0	3.5-9.1×10^3^/μL
Neutrophils	49.7	44.0-72.0%
Lymphocytes	31.8	18.0-59.0%
Monocytes	13.2	0.0-12.0%
Eosinophils	3.7	0.0-10.0%
Basophils	1.6	0.0-3.0%
Red blood cells	3.57	3.76-5.50×10^6^/μL
Hemoglobin	10.9	11.3-15.2 g/dL
Hematocrit	33.2	33.4-44.9%
Mean corpuscular volume	92.8	79.0-100.0 fl
Platelets	33.7	13.0-36.9×10^4^/μL
Total protein	6.7	6.5-8.3 g/dL
Albumin	3.3	3.8-5.3 g/dL
Total bilirubin	0.5	0.2-1.2 mg/dL
Aspartate aminotransferase	15	8-38 IU/L
Alanine aminotransferase	8	4-43 IU/L
Alkaline phosphatase	102	106-322 U/L
γ-Glutamyl transpeptidase	15	<48 IU/L
Lactate dehydrogenase	225	121-245 U/L
Blood urea nitrogen	13.4	8-20 mg/dL
Creatinine	0.60	0.40-1.10 mg/dL
eGFR	69.8	>60.0 mL/min/L
Serum Na	136	135-150 mEq/L
Serum K	5.3	3.5-5.3 mEq/L
Serum Cl	102	98-110 mEq/L
Serum Ca	9.5	8.8-10.2 mg/dL
CK	30	56-244 U/L
CRP	1.37	<0.30 mg/dL
SARS-CoV-2 antigen	-	
Urine test		
Leukocyte	Negative	Negative
Nitrite	Negative	Negative
Protein	Negative	Negative
Glucose	Negative	Negative
Urobilinogen	Negative	Negative
Bilirubin	Negative	Negative
Ketone	Negative	Negative
Blood	Negative	Negative
pH	7.5	
Specific gravity	1.015	

**Figure 1:**
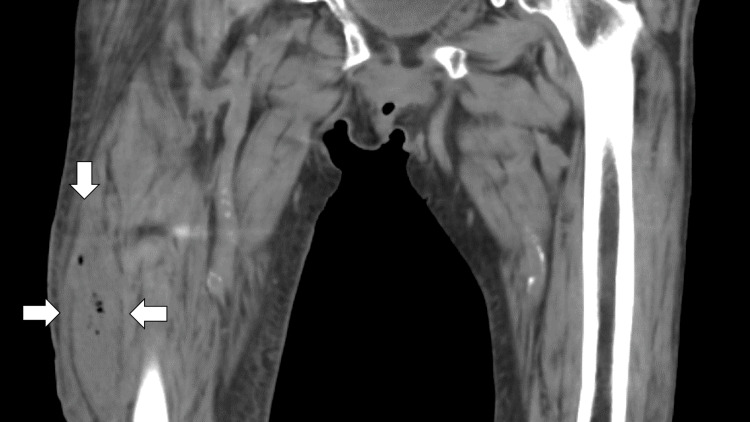
Femoral CT showing fluid retention and the included gas in the lateral part of the right thigh connecting with the previous surgical site of the femoral neck fracture (white arrows) CT: computed tomography

Femoral neck X-ray showed no fractures, but computed tomography (CT) revealed fluid and gas in the lateral right thigh, connected to the previous surgical site (Figure 1).

Aspiration of the fluid yielded pus, and the gram stain showed multiple neutrophils and gram-positive cocci. Suspecting MRSA surgical site infection, we consulted an orthopedic surgeon. The patient underwent surgical drainage for an abscess in the right thigh and was started on vancomycin (1 g daily) intravenously. Cultures confirmed MRSA, but echocardiography showed no valvular vegetation. Vancomycin was continued for two weeks post-negative blood culture results. During this period, she developed acute heart failure and hospital-acquired pneumonia and was treated with furosemide (40 mg daily) and tazobactam/piperacillin (13.5 g daily) intravenously for one week. On day 57, due to recurrent MRSA infection, her treatment was changed to monomycin and rifampicin orally.

**Figure 2:**
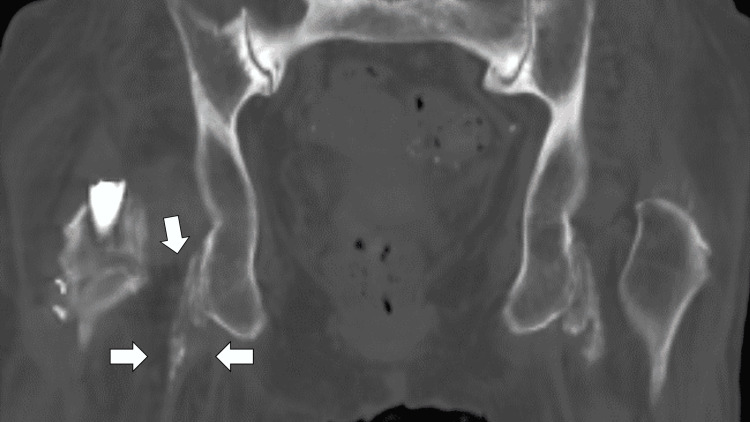
Femoral CT showing multiple migratory calcifications around the right ischial tuberosity surrounding the edematous change of the soft tissues (white arrows) CT: computed tomography

On day 67, the patient experienced acute pain from the right thigh to the hip, with a physical examination revealing tenderness and swelling in the right hip. Laboratory data showed mildly elevated C-reactive protein (CRP) and thrombocytosis. Femoral CT did not indicate abscess formation but revealed multiple migratory calcifications around the right ischial tuberosity with associated soft tissue edema (Figure 2). Initially suspecting MRSA reinfection, we started vancomycin. However, negative blood cultures for a week and CT findings led to a diagnosis of migratory pseudogout at the right ischial tuberosity (Figure 2).

Treatment with etodolac (400 mg daily) for one week improved her symptoms, and she was subsequently transferred to the rehabilitation unit on day 80.

## Discussion

The presented case report is a significant contribution to the field of medicine, particularly in understanding the complexities surrounding post-surgical infections and the challenges in accurate diagnosis. It underscores the critical need for general physicians to remain vigilant and considerate of various possibilities when encountering patients with a history of surgical site infections, particularly those involving MRSA. This report distinctly highlights the importance of differentiating between surgical site infections and other conditions, such as migratory pseudogout, which can present with similar symptoms but require markedly different treatment approaches.

One of the key insights from this report is the necessity for general physicians to consider MRSA infections in surgical sites, even when appropriate antibiotic therapy has been administered. This awareness is crucial because MRSA infections can persist or recur, posing significant risks to older patients, especially those with multiple comorbidities [7,8]. The report emphasizes the importance of comprehensive physical examinations to accurately distinguish between infection and conditions like migratory pseudogout, which may not primarily present as joint manifestations [6]. Such meticulous assessments are imperative to avoid misdiagnosis and ensure appropriate treatment.

Furthermore, the report elucidates the need for heightened caution regarding fever in patients with a history of orthopedic surgeries and MRSA infections. In such cases, fever should prompt immediate clinical attention, considering the possibility of MRSA surgical site infection, which can develop at any time post surgery [9,10]. This point is particularly salient given the potential for such infections to present atypically in older patients or those with weakened immune systems.

The case report also brings to light the occurrence of migratory pseudogout in patients with frequent infection sites and multiple calcifications in joints and tissues around bones. This correlation is vital for clinicians to understand, as it guides them to consider migratory pseudogout as a differential diagnosis in patients presenting with frequent infections, especially in systemic soft tissues [11,12]. Recognizing this relationship is essential in guiding clinical decision-making and ensuring patients receive the most appropriate care.

Finally, the report advocates for the immediate initiation of infection treatment post-physical examination and reconsidering the potential for migratory pseudogout. This approach is crucial for effective diagnosis and treatment [13,14]. It also highlights the importance of avoiding the inappropriate use of antibiotics, which can lead to antibiotic resistance and other complications [15]. The case report serves as a valuable reminder for general physicians to maintain a broad differential diagnosis and approach each case with high clinical suspicion and thorough investigation [16]. This will enable the delivery of tailored and effective patient care, improving outcomes in complex cases such as the one described.

## Conclusions

This case report emphasizes the critical need for general physicians to maintain vigilance and a broad differential diagnosis when managing post-surgical complications, especially in MRSA infections and migratory pseudogout. It highlights the importance of considering MRSA in surgical sites post-antibiotic therapy, thorough physical examinations for accurate diagnosis, and distinguishing between infection and conditions like migratory pseudogout. The report advocates for prompt and appropriate treatment of infections and careful consideration of migratory pseudogout, underscoring the importance of avoiding unnecessary antibiotic use and ensuring effective patient care.
